# Change in tooth mobility following non‐surgical periodontal therapy: A retrospective cohort study of clinical outcomes

**DOI:** 10.1002/jper.70046

**Published:** 2026-01-19

**Authors:** Georgios S. Chatzopoulos, Larry F. Wolff

**Affiliations:** ^1^ Division of Periodontology School of Dentistry University of Minnesota Minneapolis Minnesota USA

**Keywords:** electronic health records, non‐surgical periodontal therapy, periodontitis, prognosis, scaling and root planing, tooth mobility

## Abstract

**Background:**

To evaluate the change in clinical tooth mobility following scaling and root planing (SRP) and to identify baseline factors predictive of the 12‐month outcome.

**Methods:**

This retrospective cohort study utilized de‐identified electronic health records from the BigMouth Dental Data Repository. The final cohort consisted of 152 patients, contributing 489 teeth with baseline mobility of Class 1, 2, or 3. The primary outcome was the change in mobility class at 12 months. A multilevel cumulative link model (ordinal logistic regression) was used to determine the association between baseline factors (including splinting status) and the 12‐month mobility outcome.

**Results:**

Scaling and root planing resulted in a substantial reduction in tooth mobility. At 12 months, 71.2% of teeth with initial Class 1 mobility and 42.2% of teeth with initial Class 2 mobility became clinically stable (Class 0). The multilevel regression analysis identified several factors significantly associated with higher odds of a less favorable outcome: higher initial mobility, deeper probing depths, furcation involvement, smoking, and diabetes (*p* < 0.05). The presence of a splint/stabilization was significantly associated with higher odds of a more favorable mobility outcome (OR = 2.15, *p* < 0.01).

**Conclusions:**

Within the limitations of this retrospective study, SRP appears effective in reducing tooth mobility within 1 year. The identified predictors can help clinicians to manage patient's expectations and to highlight cases that may require more intensive therapy or monitoring.

**Plain language summary:**

Severe gum disease can cause teeth to become loose, putting them at risk of being lost. This study investigated whether a common “deep cleaning” procedure (non‐surgical periodontal therapy) could help to stabilize these loose teeth. We analyzed the de‐identified dental records of 152 patients from eight U.S. university clinics, tracking the outcomes of 489 loose teeth for 1 year after treatment. Our results showed that the deep cleaning was very successful. Most teeth, even those that were moderately or severely loose, became significantly firmer. For example, more than 70% of slightly loose teeth became completely stable again. We also identified factors that made a successful outcome less likely, including smoking, diabetes, having more severe gum disease, or having a very loose tooth to begin with. This research provides evidence that this routine therapy is effective in tightening loose teeth, which can help dentists and patients to make better‐informed decisions about saving teeth and maintaining oral health.

## 1 INTRODUCTION

Periodontitis is a chronic inflammatory disease initiated by a dysbiotic subgingival biofilm, leading to the progressive destruction of the tooth‐supporting apparatus.^1^ A primary clinical sign of this destructive process is increasing tooth mobility, which reflects the loss of alveolar bone and periodontal ligament integrity. This mobility can be exacerbated by excessive occlusal forces, a condition known as occlusal trauma, which in the presence of inflammation may accelerate attachment loss and threaten the long‐term prognosis of the dentition.^2^ A recent systematic review and meta‐analysis confirmed that baseline tooth mobility is a significant predictor for tooth extraction and loss, with the risk increasing proportionally with the severity of mobility.^3^ Consequently, understanding and managing tooth mobility is a central objective in periodontal therapy, aiming to improve patient comfort, function, and the overall longevity of the natural dentition.

The foundational approach to managing periodontitis is non‐surgical periodontal therapy (NSPT), which involves the mechanical removal of bacterial plaque and calculus to control inflammation. The effectiveness of NSPT in improving clinical parameters such as probing depth (PD) and achieving pocket closure has been well documented, even in the most severe cases of periodontitis.^4^, ^5^ However, the outcomes of NSPT are not uniform and can be influenced by a multitude of factors at the patient, tooth, and site level.^6^, ^7^ These studies demonstrate that while NSPT is a cornerstone of treatment, its success is variable and dependent on initial clinical conditions and patient‐specific factors.

Tooth mobility is consistently identified as a critical prognostic indicator for long‐term tooth survival. A comprehensive systematic review identified tooth mobility as one of the strongest tooth‐level predictors for tooth loss in patients with periodontitis.^8^ This is supported by numerous long‐term retrospective studies where increased mobility at the start of supportive periodontal care was significantly correlated with a higher incidence of tooth loss over many years.^9^ Despite this risk, high tooth longevity rates are achievable in well‐maintained patients, suggesting that while mobility is a significant concern, it can often be managed successfully with consistent, long‐term care.^10^, ^11^ The clinical challenge is further complicated by anatomical and functional nuances, such as furcation involvement, and co‐factors such as bruxism, which can compound the risk.^12^, ^13^, ^14^

Given the complexity of predicting periodontal outcomes, large‐scale analyses using real‐world data from electronic health records offer a powerful tool for understanding treatment effectiveness under real‐world clinical circumstances.^15^, ^16^ While the link between tooth mobility and negative long‐term outcomes such as tooth loss is well established,^17^, ^18^ and its value as a diagnostic indicator is recognized,^19^ a significant knowledge gap remains regarding the specific trajectory of mobility improvement following NSPT. Building on foundational work that identified key predictors for long‐term outcomes,^20^ this study utilized a clinical dataset to address specifically the following specific aims. The primary aim of this retrospective study was to evaluate the change in clinical tooth mobility following scaling and root planing in a multi‐center cohort. A secondary aim was to identify key patient‐ and tooth‐level baseline factors that predict the treatment outcome 1 year after therapy.

## 2 MATERIALS AND METHODS

### 2.1 Study population and data source

This retrospective cohort study was conducted in accordance with the Helsinki Declaration of 1975, as revised in 2013. The study protocol was reviewed by the Institutional Review Board of the University of Minnesota and was determined not to constitute research involving human subjects (STUDY00016576). Further approval was granted by the BigMouth Consortium for Oral Health Research and Informatics. Data were sourced from the BigMouth Dental Data Repository, which aggregates de‐identified electronic health records (EHRs) from eight contributing U.S. university dental clinics. The study utilized records entered between 2011 and 2021 by numerous dental students, residents, and faculty providers. The following university dental clinics contributed data: Harvard University, University of Texas Health, The University of California, San Francisco, University of Colorado, Loma Linda University, University of Buffalo, The University of Iowa, and The University of Minnesota. The study followed the Strengthening the Reporting of Observational Studies in Epidemiology (STROBE) and the REporting of studies Conducted using Observational Routinely‐collected health Data (RECORD) statement guidelines.^21^, ^22^

**FIGURE 1:**
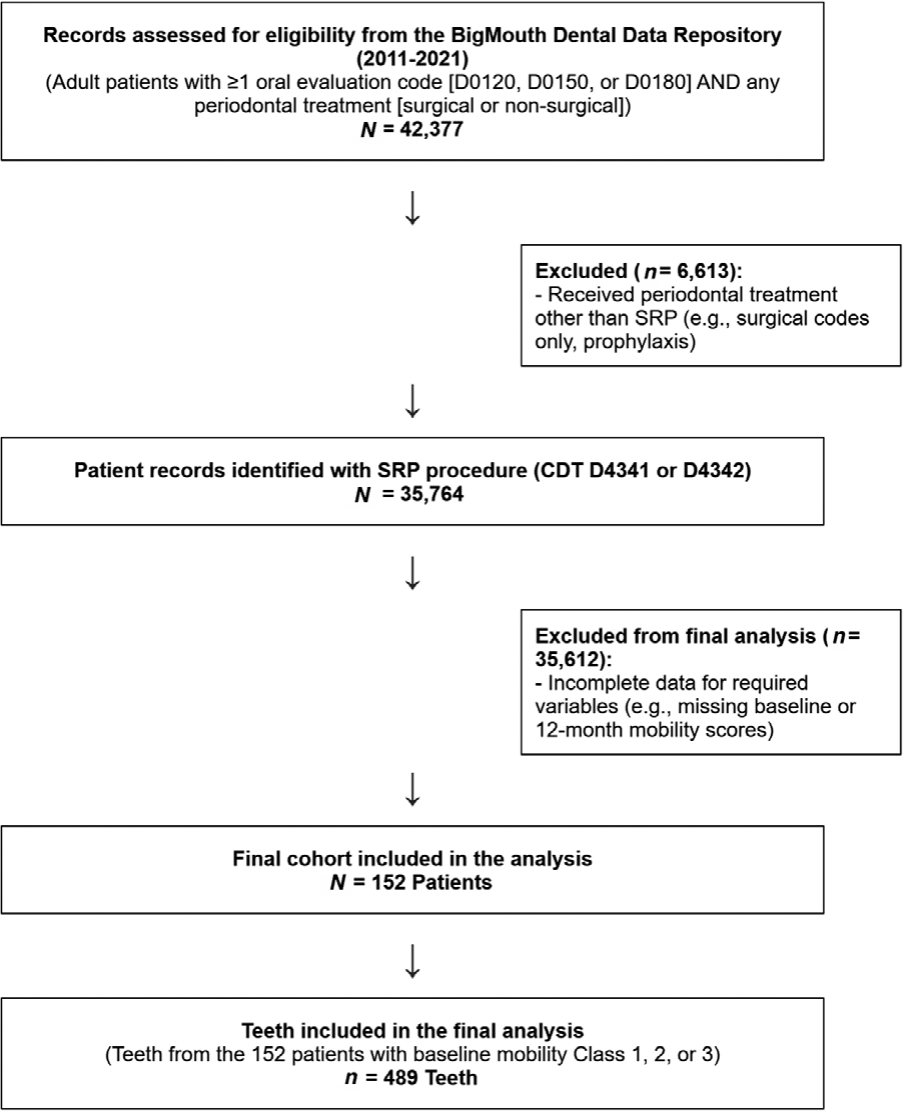
STROBE flowchart of patient selection from the BigMouth Dental Data Repository.

The patient selection process is illustrated in Figure 1. A patient was defined as having periodontitis if they received treatment codes for scaling and root planing (SRP), supported by baseline clinical parameters (mean PD ≥ 4.0 mm) consistent with a diagnosis requiring such therapy. The study population was selected from adult patients who had received at least one comprehensive or periodic oral evaluation (Current Dental Terminology (CDT), a standardized code set for reporting dental services, codes D0150, D0120, or D0180). From this initial pool, the final cohort was identified as patients with a recorded procedure for SRP for four or more teeth per quadrant (CDT D4341 or D4342). As this was a retrospective study utilizing a convenience sample from a large data repository, a formal a priori sample size calculation was not performed; the sample size was determined by the number of available records meeting the eligibility criteria. The date of the first recorded SRP procedure was used as a pragmatic and consistent baseline (T0) for longitudinal analysis.

### 2.2 Data extraction and variables

Data extraction from the EHRs focused on variables relevant to predicting changes in periodontal health. Patient‐level data included demographic characteristics (age, sex) and self‐reported systemic health conditions, such as diabetes (without data on glycemic control) and smoking status (defined as “current smoker” versus “non‐smoker”, which included former smokers). Tooth‐specific data were extracted from periodontal charting records. Key variables included probing depth (PD), clinical attachment loss (CAL), and furcation involvement (recorded as a binary variable (present/absent) for multi‐rooted teeth). Tooth mobility was treated as an ordered categorical variable, recorded as Class 0 (no mobility), Class 1 (slight mobility), Class 2 (moderate mobility), or Class 3 (severe mobility).^23^ Data regarding prosthetic status (e.g., abutments for fixed or removable prostheses) and the presence of splinting were extracted from clinical notes and procedure codes where available. Splinting status was recorded as a binary variable (present/absent) for the final analysis.

To assess the outcome of scaling and root planing, tooth mobility scores were tracked from the baseline examination immediately preceding SRP to follow‐up examinations occurring at approximately 12 months. The primary outcome was the change in mobility class for each tooth over the 12‐month period. While non‐surgical periodontal therapy (NSPT) is a broad term, for the purposes of this study, the specific and codifiable CDT codes for scaling and root planing (SRP) were used as the operational definition of the primary therapy investigated.

### 2.3 Statistical analysis

All data processing and statistical analyses were performed to evaluate the change in tooth mobility following SRP. The analysis was conducted on cases with complete data for the required variables at baseline and the 12‐month follow‐up. Descriptive statistics were used to summarize the cohort's characteristics. The primary outcome was assessed using a mobility transition matrix, with Chi‐square tests used to determine the significance of the observed changes. To account for the hierarchical nature of the data (teeth nested within patients), a multilevel cumulative link model (proportional odds logistic regression) was used to identify significant predictors of the 12‐month mobility score as an ordered categorical outcome. A random intercept for each patient was included in the model to account for the non‐independence of teeth from the same individual. The model assessed the association between baseline factors (initial mobility class, initial PD, smoking, diabetes, furcation involvement) and the final 12‐month mobility outcome, providing adjusted odds ratios (ORs) and 95% confidence intervals (CI). The proportional odds assumption was checked, and potential multicollinearity among predictor variables was assessed using the variance inflation factor (VIF). For the “initial mobility” variable, Class 1 was used as the reference category, as all teeth in the analytic cohort had baseline mobility of Class 1 or higher. A *p*‐value of < 0.05 was considered statistically significant. All statistical analyses were performed using R software (version 4.3.1, R Foundation for Statistical Computing, Vienna, Austria).

## 3 RESULTS

The patient selection process resulted in a final study cohort of 152 patients with a mean of 24.3 (± 4.1) teeth present at baseline. Within this patient group, the analysis focused specifically on teeth that presented with clinical mobility at the baseline examination, resulting in a total of 489 teeth with baseline mobility scores of Class 1, 2, or 3, which were tracked over a 12‐month period.

The cohort demographics and baseline clinical characteristics are shown in Table 1. The study cohort had a mean age of 58.4 years, was predominantly female (54%), and included a significant proportion of patients with key systemic risk factors, with 30% identified as smokers and 23% having a diagnosis of diabetes. At baseline, the 489 teeth included in the analysis showed a mobility distribution where the majority were Class 1 (56%), followed by Class 2 (33%), and Class 3 (11%).

**TABLE 1:** Cohort demographics and baseline clinical characteristics.

Characteristic	Value
**Number of patients analyzed**	**152**
**Number of teeth with mobility**	**489**
**Sex**	
Male	70 (46%)
Female	82 (54%)
**Systemic factors**	
Smokers	45 (30%)
Diabetic	35 (23%)
**Baseline periodontal status**	
Mean PD (SD)	4.1 mm (± 1.3 mm)
Mean CAL (SD)	4.9 mm (± 1.6 mm)
**Baseline mobility distribution**	
Class 1	274 teeth (56%)
Class 2	161 teeth (33%)
Class 3	54 teeth (11%)

Abbreviations: CAL, clinical attachment loss; PD, probing depth; SD, standard deviation.

The clinical track of the teeth from their baseline mobility class to their outcome at the 12‐month follow‐up is shown in Table 2. The transition in mobility status from baseline to the 12‐month follow‐up revealed a high degree of success. For teeth starting with the slightest mobility (Class 1), 71.2% (195 teeth) became clinically stable (Class 0). Teeth with moderate mobility (Class 2) also showed remarkable improvement, with 86.3% improving to a lower mobility class. Even teeth with the most severe initial mobility (Class 3) responded favorably, with over 61% improving to a less mobile state. The progression to a worse mobility class was rare.

**TABLE 2:** The clinical track of the teeth from their baseline mobility class to their outcome at the 12‐month follow‐up, with *p*‐values from Chi‐square tests.

Baseline class	Became stable (Class 0)	Class 1	Class 2	Class 3	*p*‐value
**Class 1** (274 teeth)	195 (71.2%)	73 (26.6%)	6 (2.2%)	0 (0.0%)	<0.001
**Class 2** (161 teeth)	68 (42.2%)	71 (44.1%)	20 (12.4%)	2 (1.2%)	<0.001
**Class 3** (54 teeth)	11 (20.4%)	22 (40.7%)	18 (33.3%)	3 (5.6%)	<0.001

*Note*: *p*‐values represent the significance of the change from baseline distribution for each initial mobility class, as determined by Chi‐square tests.

The predictors of a less favorable mobility outcome are shown in Table 3. The multilevel ordinal logistic regression model identified several factors that were statistically significant predictors of having higher odds of being in a worse mobility class at 12 months. Higher initial mobility, deeper probing depths, furcation involvement, smoking, and diabetes were all associated with a poorer outcome. In contrast, the presence of a splint/stabilization was a strong and statistically significant predictor of a better outcome, associated with more than double the odds of achieving a more favorable mobility class at 12 months.

**TABLE 3:** Multilevel ordinal logistic regression model of predictors for the 12‐month mobility outcome.

Baseline factor	Odds ratio (OR)	95% confidence interval	*p*‐value
**Initial mobility (ref: class 1)**			
Initial mobility class 2 vs 1	2.85	(1.90–4.28)	<0.001
Initial mobility class 3 vs 1	6.10	(3.55–10.4)	<0.001
**Initial probing depth (per mm)**	1.22	(1.10–1.35)	<0.001
**Furcation involvement (present)**	1.98	(1.25–3.14)	<0.01
**Smoking status (smoker)**	1.75	(1.09–2.81)	<0.05
**Diabetes status (diabetic)**	1.68	(1.02–2.77)	<0.05
**Splinting status (splinted/stabilized)**	0.47	(0.28–0.79)	<0.01

*Note*: An OR > 1 indicates higher odds of a worse outcome. An OR < 1 indicates lower odds of a worse outcome (i.e., a better outcome).

## 4 DISCUSSION

This retrospective cohort study aimed to evaluate the change in clinical tooth mobility following SRP and to identify baseline predictors of the 12‐month outcome. Scaling and root planing was found to be effective at reducing tooth mobility, with a substantial proportion of teeth improving to a lower mobility class or becoming clinically stable. As a secondary outcome, the analysis identified several statistically significant predictors of a less favorable mobility outcome: higher initial mobility class, greater initial probing depths, furcation involvement, smoking, and a diagnosis of diabetes.

The main strength of this study lies in its use of a multi‐center dataset, which enhances the external validity of our findings. Additionally, our multilevel model provided adjusted estimates by considering all predictor variables simultaneously, which helps to account for their potential confounding effects. However, the study has several limitations inherent to its retrospective design. Data collection relied on existing electronic health records from multiple providers without inter‐examiner calibration, which introduces a potential for information bias and limits the reproducibility of the mobility assessment. Additionally, the case definition for periodontitis used for cohort selection was based on pragmatic criteria available in the EHR data (receipt of SRP codes and mean PD). While necessary for this type of research, this definition may not perfectly align with the current 2017 World Workshop Classification for Periodontitis, which is a recognized limitation of using routinely collected clinical data.

It is critical to acknowledge that the 12‐month follow‐up period, while sufficient to demonstrate short‐term efficacy, does not provide insight into the long‐term stability of the observed improvements, which is a significant limitation. Furthermore, the dataset lacked information on several key variables. While we were able to account for the presence of splinting, the dataset still lacked detailed information on the type of splint, its duration, and the presence of occlusal adjustments, which remain as unmeasured confounders. Data on patient compliance, race/ethnicity distribution and occlusal factors were not available for analysis. The absence of occlusal and prosthetic data is particularly noteworthy, as persistent occlusal trauma or prosthetic loading could act as significant confounding variables limiting the therapeutic effect of inflammation control alone.^2^

Our findings are well supported by the existing body of evidence. The observation that controlling inflammation through SRP leads to a reduction in tooth mobility aligns with the fundamental biological principles of periodontal therapy. The likely mechanism is the resolution of inflammation within the periodontal ligament, which reduces edema and enzymatic degradation of collagen fibers, allowing for tissue repair and a clinical stability of the tooth.^24^, ^25^, ^26^, ^27^ The negative impact of smoking and diabetes on this healing process is also well established, as these conditions impair microvascular function and the host immune response.^28^, ^29^ Similarly, the finding that furcation involvement on multi‐rooted teeth was a strong negative predictor aligns with previous literature, as these anatomical features present significant challenges for both therapy and long‐term maintenance.^8^, ^9^ While no single systematic review focuses exclusively on the improvement of mobility as an outcome, our results on the predictors of a poor outcome are strongly corroborated by systematic reviews that identify these same factors (initial mobility, smoking, diabetes, furcation involvement) as significant risks for the ultimate negative endpoint: tooth loss.^3^, ^8^, ^9^

This study adds a crucial perspective to the available evidence by quantifying the potential for mobility reduction after SRP, even after controlling for the powerful stabilizing effect of adjunctive splinting. While much of the literature focuses on predicting irreversible tooth loss,^9^, ^10^, ^12^ our results quantify the significant potential for stabilization of mobile teeth through conventional NSPT.^4^, ^5^, ^6^ This has direct implications for patient care and communication. While rare, the progression of a few teeth to a worse mobility class warrants attention. This may reflect sites with refractory disease or the influence of unmeasured confounders such as occlusal trauma, underscoring the need for careful monitoring during follow‐up. For health policy, this evidence reinforces the value of SRP as a primary intervention for preserving the natural dentition and maintaining function, supporting its coverage and promotion as a cornerstone of oral healthcare.

The results of this study also touch upon existing clinical controversies, particularly regarding the management of teeth with moderate to severe mobility. While our data show that even Class 2 and 3 mobile teeth can improve with SRP alone, a substantial portion do not achieve complete stability. This fuels the ongoing debate about the necessity and timing of adjunctive therapies, such as occlusal adjustment or splinting.^2^ Data on patient compliance, race/ethnicity distribution, occlusal factors, and prosthetic data were not available for analysis. The absence of occlusal and prosthetic data is particularly noteworthy, as persistent occlusal trauma or prosthetic loading could act as significant confounding variables limiting the therapeutic effect of inflammation control alone.^2^ The findings highlight the need for a multifactorial approach to diagnosis and treatment planning that considers both the inflammatory and biomechanical aspects of tooth mobility. Finally, while our statistical model is robust for this data structure, it serves as an important exploratory analysis; future research could benefit from more detailed diagnostic checks of model fit and assumptions.

Several well‐established predictors of periodontal disease progression, such as clinical attachment loss (CAL) and tooth type, were considered during the initial stages of our statistical modeling. Given that tooth mobility is fundamentally a consequence of lost periodontal support, clinical attachment loss was expected to be a strong predictor. However, a preliminary analysis revealed a high degree of collinearity between baseline CAL and baseline probing depth (PD) in our dataset. To avoid issues with multicollinearity and to create a more parsimonious and stable predictive model, PD was retained as the primary measure of site‐specific periodontal destruction, and CAL was subsequently excluded from the final multivariable model. Similarly, while tooth type is often associated with different prognoses, particularly for molars, it was not found to be a statistically significant predictor of the 12‐month mobility outcome in our final adjusted multilevel model after accounting for the effects of initial mobility, probing depth, and furcation involvement. Therefore, the final model presented focuses on the most significant and independent predictors identified in our analysis.

Based on these findings, several avenues for future research are apparent. Prospective longitudinal studies with follow‐up periods extending beyond 1 year are needed to determine the long‐term stability of the improvements observed after SRP. Future clinical research should aim to incorporate detailed occlusal analysis to clarify the relative contributions of inflammation and occlusal trauma to tooth mobility and its resolution, especially in patients with co‐factors such as bruxism.^14^ Furthermore, understanding why certain sites remain refractory to treatment is critical.^13^

## 5 CONCLUSIONS

Within the limitations of this retrospective analysis, this study demonstrates that scaling and root planing is an effective intervention for reducing clinical tooth mobility over a 12‐month period. A significant proportion of teeth can achieve clinical stability. The identification of baseline predictors—namely, higher initial mobility, deeper probing depths, furcation involvement, smoking, and diabetes—provides clinicians with valuable prognostic information, underscoring the importance of a comprehensive approach to patient care. These factors underscore the importance of a comprehensive approach that addresses both local periodontal inflammation and systemic patient‐level risks to optimize the potential for periodontal healing and the preservation of the natural dentition

## AUTHOR CONTRIBUTIONS

Both authors have made substantial contributions to the work. **Georgios S. Chatzopoulos**: contributed to the conception and design of the work and the acquisition of data; analysis and interpretation of data for the work and for drafting the manuscript; final approval of the version to be published. **Larry F. Wolff**: contributed to the conception and design of the work and the acquisition of data; revised the work critically for important intellectual content; gave final approval of the version to be published. Both authors agree to be accountable for all aspects of the work in ensuring that questions related to the accuracy or integrity of any part of the work are appropriately investigated and resolved.

## CONFLICT OF INTEREST STATEMENT

The authors declare no conflicts of interest.

## Data Availability

The data that support the findings of this study are available from the corresponding author upon reasonable request.
